# The Genetic Origin of Uneven Cognitive Profiles in Heritable Neurodevelopmental Conditions and Individual Differences: Computational Investigations

**DOI:** 10.1111/desc.70186

**Published:** 2026-04-16

**Authors:** Maitrei Kohli, George Magoulas, Michael S. C. Thomas

**Affiliations:** ^1^ Department of Computer Science University College London London UK; ^2^ School of Computing and Mathematical Science Birkbeck University of London London UK; ^3^ Developmental Neurocognition Lab, Centre for Brain and Cognitive Development, School of Psychological Sciences Birkbeck University of London London UK

**Keywords:** artificial neural networks, dyslexia, evolutionary selection, genetics, heritability, neurodevelopmental conditions, uneven cognitive profiles

## Abstract

While the heterogeneity and co‐occurrence of heritable neurodevelopmental conditions such as autism, attention deficit hyperactivity disorder (ADHD), and dyslexia remain issues of debate, these conditions are nevertheless all characterised by uneven cognitive profiles exhibiting strengths and weaknesses. There have been advances in understanding neural markers and genetic predictors of these conditions, but little insight into how DNA variation can influence functional brain development in such a way as to produce uneven cognitive profiles as developmental outcomes. Uneven cognitive profiles (e.g., across verbal and non‐verbal intelligence) also characterise individual differences, and similarly, their genetic basis is little understood. Two main sources of uneven profiles appear possible: that there are regional genetic effects on brain development that act on mechanisms which play an influential role in the development of a particular cognitive or socioemotional ability (domain‐specificity); or that genetic effects on brain development have a more widespread influence on neurocomputational properties, but the development of particular abilities is differentially sensitive to variation in those properties (domain‐relevance). In this article, we present computational simulations that combine genetic algorithms and artificial neural networks to explore the second of these possibilities, domain relevance. Selection is used to alter the population frequency of alleles that influence the neurocomputational properties of a common substrate, under a polygenic model. Different regions of the substrate become specialised for developing different functions, modelled by five tasks. Across 20 generations, we assess how selection for a given task, which serves to tune substrate‐wide neurocomputational properties in favour of this task, serves to alter the development of the other four tasks, which must employ the same range of neurocomputational properties. We demonstrate that such selection can enhance or impair acquisition in non‐selected domains, depending on the computational demands of each task domain. We also show that behavioural deficits associate with an increase in the heritability of individual differences. We discuss the results in the context of contemporary theories of the influence of genetic variation on functional and structural brain development, and assess the merits of the domain‐specific and domain‐relevant accounts of uneven cognitive profiles in neurodevelopmental conditions and individual differences.

**Summary:**

- Heritable neurodevelopmental conditions such as dyslexia, attention deficit hyperactivity disorder, autism, developmental language disorder, and developmental coordination disorder are characterised by uneven cognitive profiles. However, little is known about how genes produce such uneven cognitive profiles. It is a puzzle because genetic effects on brain development are typically more widespread than areas showing functional specialisation in adults.
- The work presents computational modelling to demonstrate how the relationship between cognitive domains and processing properties of a substrate constrains behavioural development. A common substrate for different domains (such as association cortex in a cortical lobe), where domains are specialised to different regions with shared properties, may exhibit uneven cognitive profiles if the processing properties of the substrate are better tuned to supporting some domains than others (so‐called domain‐relevance).
- Simulations then test the hypothesis that domain relevance is a plausible mechanism for explaining how common genetic variation might contribute to specific and uneven cognitive profiles seen in neurodevelopmental conditions.
- The computational simulations therefore give insight into how conditions such as dyslexia may emerge, why they would be heritable, and why the relationship between genotype and phenotype in common neurodevelopmental conditions is likely to be highly polygenic.

## 1 Introduction

How could dyslexia, a developmental problem with learning to read, be heritable? Found in up to 10% of children, it has been reported that around 70% of the risk of dyslexia is predicted by genetic similarity (Erbeli et al. 2021). What genes could be relevant to the recent cultural invention of linking visual symbols to speech sounds and why should such genetic variation exist?

If we follow the consensus view that deficits in phonological processing are a key contributor to difficulties in learning to read (Carroll et al. 2025), presumably the answer is as follows. Human populations had a pre‐existing, genetically influenced distribution of phonological processing skills, which prior to the invention of literacy, merely caused individuals at the lower end to have slightly slower lexical retrieval, while at the upper end perhaps a gift for learning multiple languages. Upon the invention of reading, individuals with genes predisposing them to the lower end then struggled to learn mappings between their less well segmented phonological representations and written orthographies, at least when these mappings exceeded a certain level of complexity. While some of this genetic variation has been identified (Doust et al. 2022), its role in influencing brain development and functioning is poorly understood, as is why in some cases of dyslexia, there can be a large discrepancy between reading ability and overall intellectual ability.

### 1.1 Uneven Cognitive Profiles

Uneven cognitive profiles characterise many common, behaviourally defined neurodevelopmental conditions with heritable components (e.g., attention deficit hyperactivity disorder [ADHD], autism, developmental language disorder, developmental coordination disorder). The picture is similar to dyslexia: even when DNA variation is identified that predicts the presence of these conditions, there is little understanding of how genetic influence could lead to the uneven profile (Demontis et al. 2023; Grove et al. 2019; Mountford et al. 2021; Nudel et al. 2023). While recent transdiagnostic approaches have questioned the existence of discrete neurodevelopmental conditions, instead preferring dimensional approaches (see e.g., Astle et al. 2022), nevertheless the emphasis on uneven profiles remains. For example, in a large sample of struggling child learners, Siugzdaite et al. (2020) used machine learning methods to recover uneven profiles marked by differential performance in phonological skills compared to executive function skills. They found that these profiles ran across children who had received diagnoses of conditions such as dyslexia, developmental language disorder, autism, and ADHD.

Uneven cognitive profiles are also a feature of typical development. Within the field of individual differences, a more familiar division is found between the general factor of intelligence and specific factors of intelligence. The general factor speaks to the *evenness* of cognitive profiles (correlated test scores across different cognitive abilities). Specific factors represent unevenness, whether between verbal and non‐verbal skills, or the kinds of more detailed domain strengths that have in the past led to proposals the field should consider defining ‘multiple intelligences’ (e.g., Gardner 1983). In terms of mechanistic causes, the majority of research focus has been on the general factor of intelligence, and identifying the genes that contribute to the ∼50% heritable component (Deary et al. 2022). Three kinds of causal explanation remain in play for the general factor of intelligence. First, that it reflects the operation of one or more domain‐general processing mechanisms which are involved in a range of cognitive tasks (such as the multiple demand network; Duncan 2010). Second, that it reflects developmental interactions between the multiple cognitive components required to deliver complex behaviour, interactions which serve to yoke together any intrinsic variability in the components (e.g., van der Maas et al. 2006). Or third, that it reflects variation in broad processing properties across the brain that causes the performance of separate domain‐specific systems to be correlated (e.g., Goriounova and Mansvelder 2019). The well‐supported finding that cognitive training tends only to produce only near transfer (e.g., Sala et al. 2019) argues against the widespread presence of domain‐general mechanisms in the brain, and therefore that the second or third options are the more likely cause of general intelligence.

The search for genes associated with intelligence using genome‐wide association studies has indicated high polygenicity (Reis and Spinath 2025) but has not advanced mechanistic accounts. Deary et al. (2022) concluded that the association between genetic variation and variation in intelligence is partly mediated by individual differences in gene expression across the cortex, with genetic variants tending to be linked with neurogenesis, the synapse, neuron differentiation, and oligodendrocyte differentiation. According to twin studies, specific intelligences show a high heritability of around 50%, similar to general intelligence. For example, in one sample of twins, the disparity (unevenness) between composite measures of verbal and non‐verbal intelligence showed a heritability of 40% in 12‐year‐olds (Elgamal 2026). However, a lack of genome‐wide association studies designed to predict variation in specific cognitive abilities means less is known about their possible neural basis (Procopio et al. 2022).

### 1.2 Domain‐Specific Versus Domain‐Relevant Explanations

In the context of neurodevelopmental conditions, Karmiloff‐Smith (1998) argued that there were two types of mechanistic variation that might produce uneven cognitive profiles. On the one hand there might be differences in brain processing systems *specific* to the domain showing a cognitive strength or weakness. While complex behaviours are delivered by networks of brain regions, a restriction in one region may serve as a limiting factor, leading to deficits in the development of the whole behaviour. On the other hand, there might be more widespread differences in brain processing properties, but deficits only appear in domains where the system particularly relies on those properties. In the terminology of Karmiloff‐Smith (1998), these would be processing properties that are more or less *relevant* to a given domain.

Both domain‐specific and domain‐relevant approaches can be found in explanations of neurodevelopmental conditions. For example, domain‐specific accounts of dyslexia point to under‐activation or structural differences in the temporal lobe regions involved in phonological processing. Thus, Hancock et al. (2017) proposed that the cause of dyslexia is processing noise arising from neural hyperexcitability caused by the expression of reading disorder risk genes in temporoparietal and occipitotemporal cortices. Domain‐specific accounts of ADHD point to systems involved in reward processing (a fronto‐cingulo‐striato‐thalamic network) and working memory (a fronto‐parieto‐cerebellar network) (e.g., Rubia 2018). Domain‐specific accounts of autism point to systems involved in social cognition and executive functions such as the temporoparietal junction, the insula, and dorsolateral prefrontal cortex (e.g., Jayashankar et al. 2023). By contrast, domain‐relevant studies allude to the lower IQ scores (on average) observed in dyslexia, developmental language disorder, ADHD, and autism, as well as differences in overall brain metrics such as brain size and cortical morphology (e.g., Conti‐Ramsden et al. 2012; Courchesne et al. 2003; Makowski et al. 2022; Scholz and Scheer 2020; Soheili‐Nezhad et al. 2024; Wood et al. 2011; Wolff et al. 2022). The implication is that in these neurodevelopmental conditions, there are more widespread brain differences in processing properties, which then differentially impact, respectively, the development of reading, language, attention and impulse control, and social engagement and cognitive flexibility.

The domain‐specific vs. domain‐relevant distinction is readily extended to individual differences. Specific intelligences might be explained by advantages or disadvantages in domain‐specific systems serving as limiting factors on or driving factors for specific skills (see, e.g., Ramsden et al. 2011). But they might also be explained by more widespread brain differences in properties that are particularly relevant to developing specific skills in, say, verbal or non‐verbal domains.

### 1.3 Explanatory Accounts Must Be Constrained by the Genetics of Brain Development

Biology and gene expression provide a grounding for why the domain‐relevant account is more likely than the domain‐specific account. To adjudicate between domain‐relevant and domain‐specific accounts of the heritable component of uneven cognitive profiles, we need to incorporate what we understand about the role of gene expression in the development of brain function and structure. For the domain‐specific account to be viable, there must be detailed genetic control of brain development at the regional level, corresponding to the functional areas observed to support high‐level behaviours like reading. Thus, a domain‐specific account of dyslexia that posits differences in temporal lobe regions hosting key components of the developing reading network must be predicated on there being genes whose influence on neural processing is restricted to these regions.

Brain development is an extended process (see Zhou et al. 2024, for review). It involves generation of the neural tube in the embryo and neural epithelial cell proliferation within it, neurogenesis mediated by neural stem cells, both radial and tangential migration of neural stem cells to their destination, and neural stem differentiation and maturation to establish laminar structure and areal identity. This is followed by generation of axons, dendrites and synapses to establish connectivity, and then postnatal remodelling including synaptic pruning and myelination. Marked regional differences can be observed in laminar structure, neural density, neuronal morphology and neuronal cell types, which together allow the consistent identification of cytoarchitecturally distinguishable areas such as those observed by Brodmann (Cadwell et al. 2019). Modern methods have revealed a great deal of region‐specific gene expression both developmentally and in the adult state. During development, regional differences often take the form of diffuse gradients of expression across axes such as rostral‐caudal (front‐to‐back) or medial‐lateral, offering neurons multidimensional cues to location to support the formation of their areal identities. In the adult, differences can be more abrupt and regionally specific.

For the cortex, Cadwell et al. (2019) argued that intrinsic gene expression specifies broad regional distinctions, especially between sensory and motor areas, while activity‐dependent mechanisms, particularly involving thalamo‐cortical connections, lead to progressive refinement and the formation of sharp boundaries between functional areas, which they called the *serial homology and refinement model*. The finer‐scale regional differences in gene expression are extrinsically modulated by the processes of activity‐dependent functional self‐organisation.

In short, inasmuch as genetic variation between humans influences neurocomputation, intrinsic effects appear to influence larger areas of cortex than the functional regions identified in adults, particularly outside of primary sensory and motor cortical areas.

### 1.4 The Origin of Genetic Variation Producing Uneven Cognitive Profiles

Where could genetic variation come from, for example of the sort that leads to a population distribution in phonological processing skills? There are two possibilities: it arose from evolutionary selection, or it arose from genetic drift (where drift represents stochastic changes in population gene frequency across generations).

A domain‐specific account of uneven profiles in individual differences would fit with selection if enhanced performance in a cognitive domain improved fitness. Gene variants producing stronger development in the underlying system would become more frequent. A domain‐specific account of developmental deficits would align less well with selection since, by definition, the behavioural outcome is not advantageous. The exception would be if the cultural context has changed so radically that what was once advantageous and adaptive has now become a deficit. Therefore, genetic drift seems a more likely explanation for heritable developmental deficits. So, for the example of dyslexia, in past populations, differences in skills in phonological access and phoneme manipulation might have been trivial for survival and could vary without consequence. Genetic drift accounts have been favoured for both dyslexia and ADHD from neuroscientific and molecular genetics perspectives (MacDonald et al. 2024; Mountford et al. 2025).

Domain‐relevant accounts could also appeal to genetic drift, albeit for brain properties that are more widespread. However, domain‐relevant accounts could also accommodate evolutionary selection as an explanation of changes in population gene frequency. If multiple processing systems use different regions of a substrate with common processing properties (say, across association cortex), then systems whose development is enhanced by some direction of genetic variation—sufficient to generate behaviour with a selective advantage—would lead to changes in these properties across wider regions of the substrate in future generations. All functional systems developing on the substrate would then be influenced by the new calibration of processing properties. For example, if a single system contributed to an adaptive advantage because say, in this individual, the substrate happened to have more neurons, in later generations this selective pressure would provide more neurons for all systems developing on the substrate. However, changes in domain‐relevant properties that enhance the development of some skills might impair the development of others.

A domain‐relevant account of uneven cognitive profiles would fit with the observation of more diffuse actions of gene expression across developing cortex. However, such an explanation would rely on demonstrating two points. First, that different cognitive domains require different domain‐relevant processing properties. And second, that either drift or selection of those properties to favour one cognitive domain might increase the potential for heritable developmental deficits in other domains that develop on different regions of the substrate. Proof‐of‐principle demonstration of these two points was the goal of the current work.

### 1.5 Computational Modelling

Computational modelling provides a framework within which to consider these questions in more detail. Biologically inspired artificial neural networks allow more detailed specification and evaluation of proposals about the impact of concepts like neural resources, processing noise, or plasticity on developmental outcomes. Our goal in the current work was to use computational methods to evaluate whether the domain‐relevant explanation of heritable uneven cognitive profiles is a plausible one. Such models have previously provided a bridge between putative neurocomputational processing properties and the development of high‐level behaviours characterised by cognition. Thus, various artificial neural network models have explored how reduced neurocomputational resources might generate features of dyslexia in models of reading (e.g., Seidenberg and McClelland 1989; Harm and Seidenberg 1999), or reduced plasticity might generate delays in language development (Thomas and Karmiloff‐Smith 2003; Thomas and Knowland 2014), or indeed proposed that plasticity might be a possible explanation of the general factor of intelligence in individual differences (Garlick 2002). The disadvantage of biologically inspired networks is that the simplification required for implementation means that their biological plausibility can be quite restricted, even when these models are scaled to the kinds of naturalistic behaviours generated by contemporary deep neural networks and large language models (see Thomas and McClelland 2023, for discussion).

Here we utilise a computational framework in which multiple cognitive domains were acquired across different areas of a common computational substrate. We constructed domains to embody different types of input‐output mapping problems and different levels of computational complexity. Variation in neurocomputational processing properties of the substrate was modelled as under genetic control. Population frequencies of genetic variants were then altered via selection applied to a single domain over multiple generations. We then examined the impact on development of the other non‐selected domains, and alterations to the heritability of each behaviour. We addressed two questions: Did we observe domain‐relevance? And could alterations in gene frequencies induce heritable uneven cognitive profiles?

## 2 Method

### 2.1 Overview

**FIGURE 1:**
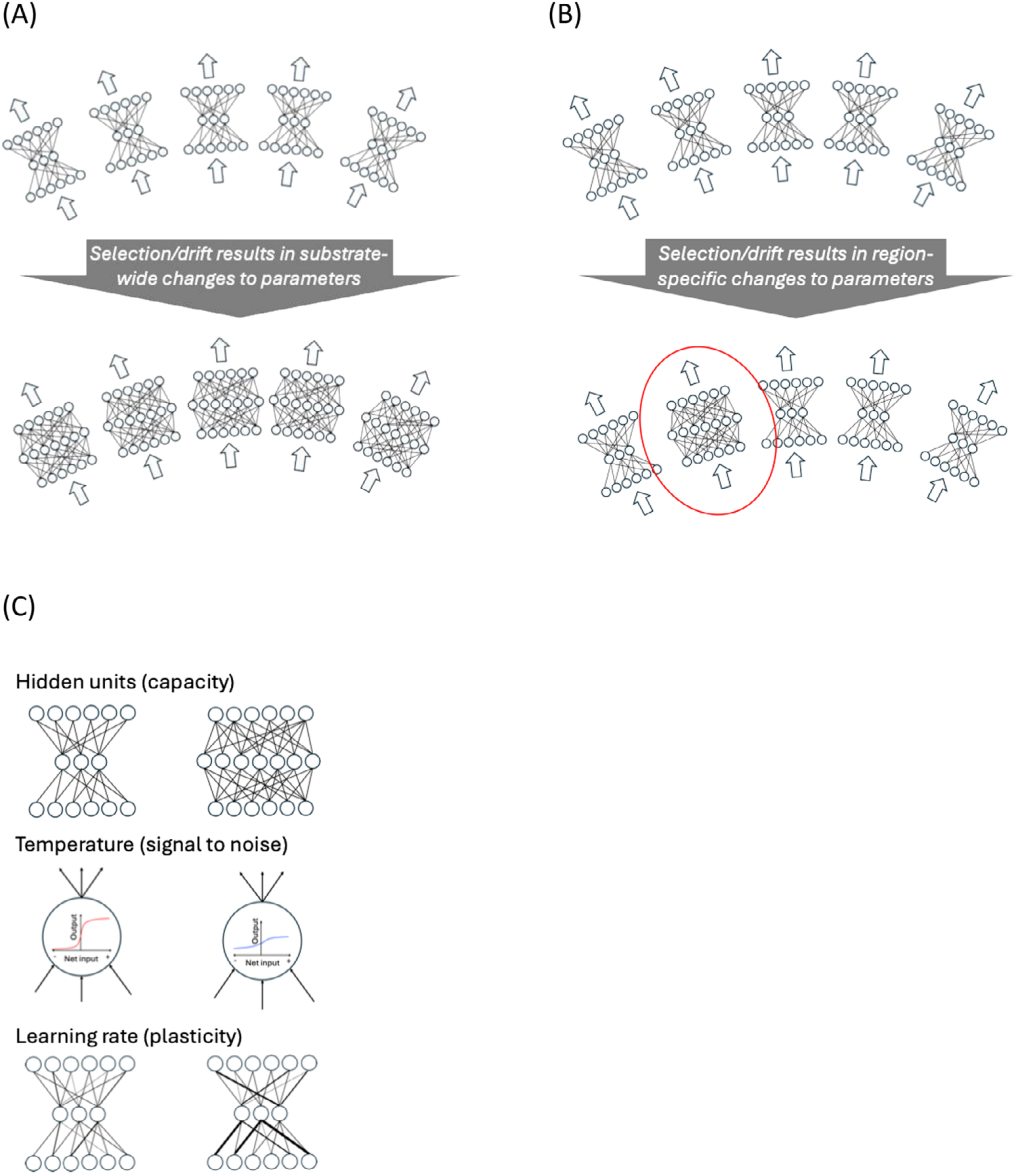
Schematic of five linked networks across a substrate, each responsible for acquiring the input‐output mappings to drive behaviour in one of five cognitive domains. (A) Domain‐relevant account: change in genetic variants causes substrate‐wide parameter changes, here in neuronal number. (B) Domain‐specific account: changes in genetic variants cause focused parameter changes in one region, here in neuronal number (outlined). (C) The three neurocomputational parameters used in the simulations to explore the impact of changes in population gene frequencies on cognitive profiles.

The simulations used population‐level modelling, simulating the development of large numbers of individuals. Five problem domains were constructed to represent different cognitive domains, each comprising a set of input‐output mappings. The domains were chosen to pose diverse computational challenges and levels of complexity to the substrate. The domains were: categorisation (CC), categorisation with exceptions (CE), quasi‐regular mapping (QR), autoassociation (AUTO), and arbitrary mapping (ARB). The acquisition of each set of mappings was taken to drive a different target behaviour. Performance on the five domains produced an individual's cognitive profile. Development occurred in five linked artificial neural networks with identical neurocomputational parameters, representing different regions across a common substrate (see Figure 1). In line with multiscale modelling approaches (e.g., Thomas et al. 2016), the neurocomputational parameters of each artificial neural network were encoded in an artificial genome, with a polygenic relationship between artificial genes and parameters. Three different neurocomputational parameters were varied across networks, specified at the beginning of the developmental process: the computational capacity (number of hidden units), the signal‐to‐noise of neural processing (the temperature of the sigmoid in each artificial neuron's activation function) and the plasticity (the learning rate constant which altered how much connection weights were altered in response to error signals). For simplicity, we did not consider parameters which changed across development, even though these are a known feature of biology (e.g., synaptic density, excitation‐inhibition balance; Huttenlocher and Dabholkar 1997; Chini et al. 2022).

To capture the heritability of individual differences in developmental outcomes, two steps were necessary. First, there needed to be both genetic and environmental contributions to variation in behaviour. Genetic variation was provided by differences in the artificial genomes which produced different neurocomputational parameter settings. Environmental variation was produced by a filter applied to the set of input‐output mappings constituting each domain as a method to capture differences in cognitive stimulation (Rakesh et al. 2024). Second, there needed to be different degrees of genetic similarity between individuals: heritability is estimated by establishing the extent to which genetic similarity predicts variation in the phenotype (here, behaviour). This was achieved by creating pairs of twins, either monozygotic twins (MZ) who shared the same genotype, or dizygotic twins (DZ), who shared 50% of genes on average. Heritability could then be estimated according to the difference in the correlations between MZ twins and between DZ twins.

**FIGURE 2:**
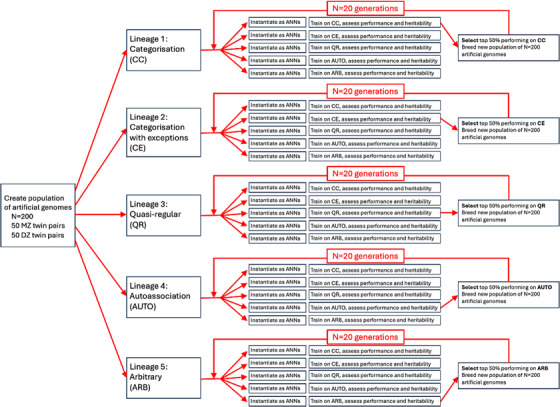
Schematic of the simulations. A population of artificial genomes is created. This is instantiated as a population of artificial neural networks (ANNs) in five separate lineages, each undergoing selection according to one of the domains. In each generation in each lineage, the new population of artificial genomes is instantiated as populations of ANNs, repeated five times to represent different areas of the substrate. Each area of the substrate is then trained on one of the domains.

As a simplification, genetic variation was modelled only by changes in gene frequency across subsequent generations of a population, rather than the introduction of genetic mutations. To induce changes in gene frequency, thereby altering the population distribution of neurocomputational parameters, we opted to use evolutionary selection. Although drift (stochastic sampling effects) would eventually produce changes over generations, selection was a more efficient method. We created five lineages, considered independently. The five lineages corresponded to selection of the substrate processing properties according to the five different cognitive domains, with the top‐performing networks allowed to sexually reproduce to found the next generation of genotypes. Selection was allowed to operate over 20 generations to alter population gene frequencies. A schematic of the simulations is shown in Figure 2. Further details of the simulation are included in Appendix A.

The modelling framework therefore encapsulated two forms of change: *evolutionary change*, as the gene frequencies alter across generations, altering the neurocomputational parameters instantiated in each new population; and *ontogenetic change*, as each instantiated substrate goes through the experience‐dependent developmental process of acquiring five functions across different regions.

We assessed three outcomes: (1) the performance change for each cognitive domain across generations, in response to selection in each lineage; (2) the change in distribution of neurocomputational parameters (mediated by selection of the relevant gene variants); and (3) the corresponding change in heritability of individual differences in the five domains.

## 3 Results

All networks underwent a developmental process. For tractability, in the results, we only consider the performance of networks at the end of this developmental process. We first established that in generation 1, prior to selection, each of the lineages demonstrated the same level of performance on the five cognitive domains. An analysis of variance on each domain including lineage as a five‐level factor confirmed no significant effect of lineage (all *p* > 0.1). Similarly, the distribution of neurocomputational parameter values in each lineage did not differ in generation 1. The five lineages therefore started off the same.

Table 1 includes the performance accuracy across the cognitive domains in generation 1. Reflecting the gradient of difficulty incorporated in the design of the training sets, performance on categorisation was the highest (and at ceiling), following by categorisation‐with‐exception (near ceiling), followed by quasi‐regular rule, autoassociation, with arbitrary association showing the lowest performance.

**TABLE 1:** Selection improved the performance of selected domains but could reduce the performance of non‐selected domains. Performance in each cognitive domain is shown at Generation 1 and at Generation 20. Outlined boxes show performance change across the generations in response to selection for that behaviour.

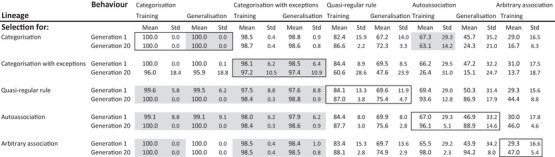

*Note*: Scores show means and standard deviations for accuracy (% correct) on training and generalisation sets. All differences between generations were reliable at *p* < 0.05 (independent samples, *t*‐test) unless shown with grey shading. Since Arbitrary association does not embody a generalisable function, there was no generalisation set.

**FIGURE 3:**
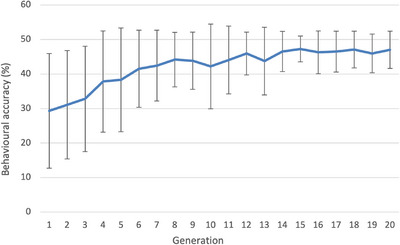
Selection improves developmental outcomes over generations. Change in population mean performance (% accuracy) on the arbitrary association domain under selection over 20 generations. Error bars show population standard deviation.

Table 1 also includes the change in performance produced by 20 generations of selection. We focus initially on performance in domains which were the target of selection (highlighted in Table 1 with a box). The categorisation tasks showed little change in performance under selection because they were near ceiling. For the other three tasks, there were reliable increases in performance across the generations on both training and generalisation sets. Figure 3 plots the increase in accuracy for arbitrary association, showing that as would be expected for a polygenically influenced trait, improvements were gradual over a number of generations, as more of the advantageous alleles were accumulated in subsequent generations.

**FIGURE 4:**
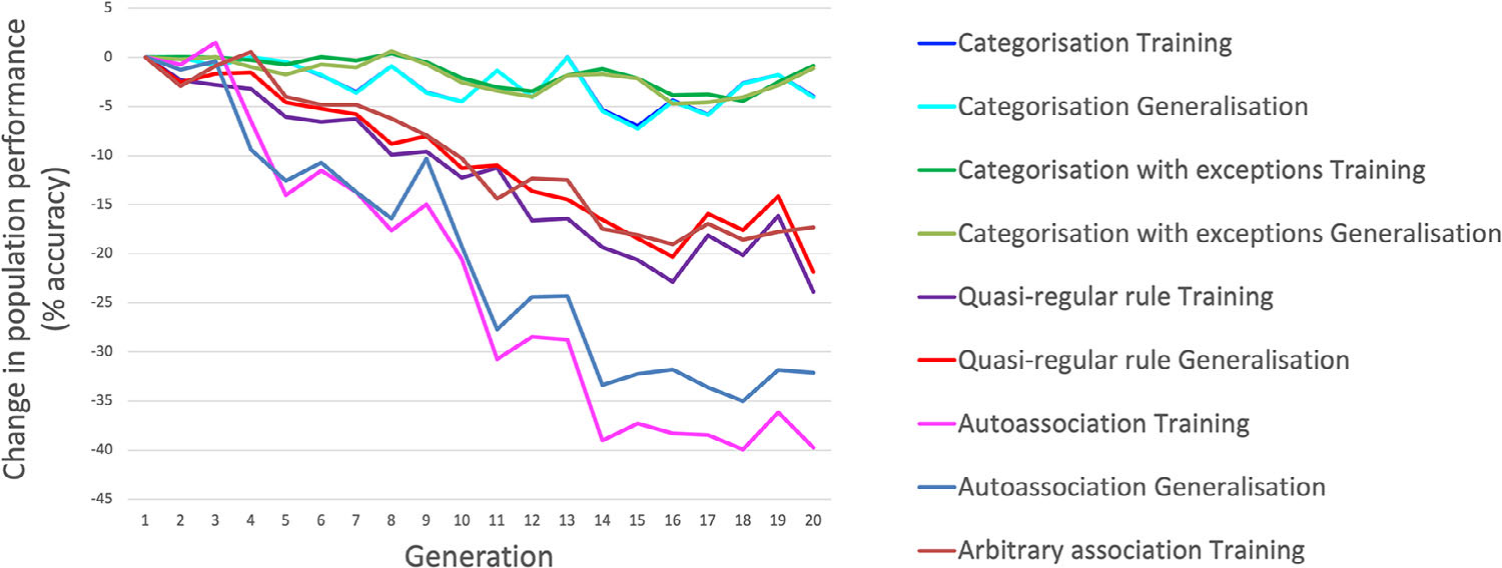
Selection can produce uneven cognitive profiles. Change in population mean performance across all behaviours over 20 generations of selection of substrate parameters favouring Categorisation with Exceptions. Profiles that were even at generation 1 have become uneven at generation 20, with strengths in categorisation domains and weaknesses in more computationally demanding domains such as arbitrary association and autoassociation.

Lastly, Table 1 includes performance levels for non‐selected domains in each lineage in generation 20. ANOVAs assessing the effects of lineage on performance change across generations showed that only with categorisation were the effects of selection absent, because the task was easy enough that it was immune to neurocomputational conditions. Selection for the computationally challenging tasks of quasi‐regular rule, autoassociation and arbitrary association had universal benefits: selection in each of these three lineages improved performance on other tasks as well, with the biggest benefit for the selected task. However, selection for the categorisation tasks produced uneven profiles. For categorisation, quasi‐regular rule improved, autoassociation was unaffected, but arbitrary association declined. For categorisation‐with‐exceptions, all other tasks declined, with the effects most marked on the most demanding tasks of autoassociation and arbitrary association. The emergence of this effect across generations is shown in Figure 4. Changes in the neurocomputational parameters under selection could therefore produce developmental impairments in other tasks with different domain‐relevant requirements.

In sum, the first demonstration of the model was that domain‐relevance can produce uneven cognitive profiles. A substrate selected to acquire one domain, such as fine‐grained classification, may simultaneously show deficits in trying to acquire another domain, such as random associations. To understand how domain‐relevance operated, we next traced the changes in the distribution of underlying neurocomputational settings induced by selection for each domain.

**FIGURE 5:**
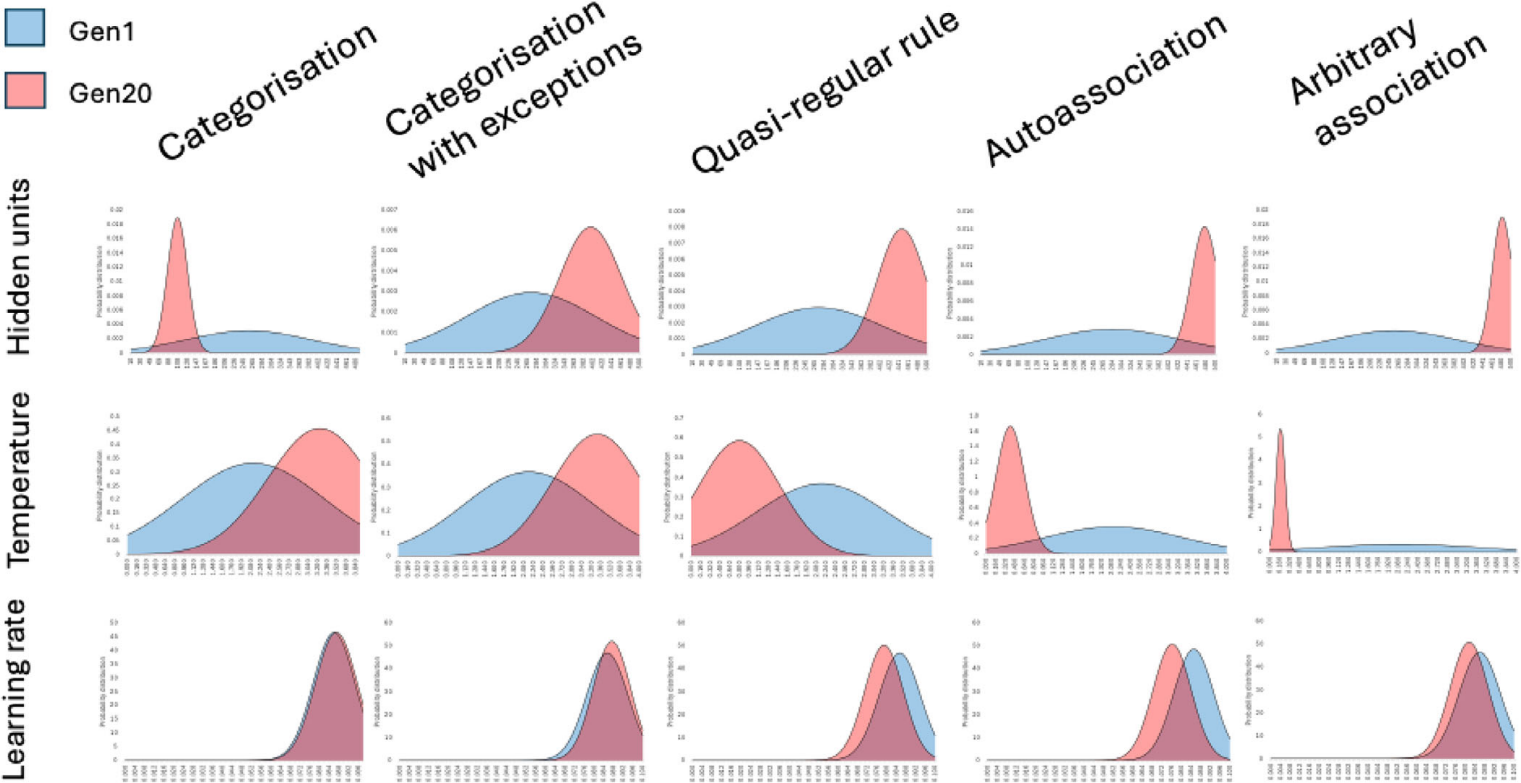
Changes in substrate parameter values as a result of selection. The three rows show the distribution of parameter values in the population for hidden units, sigmoid temperature, and learning rate (x‐axis values depend on parameter). Each row has five plots indicating what happens to the distribution of parameter values in different lineages under selection for, respectively, categorisation, categorisation with exceptions, quasi‐regular rule, autoassociation, and arbitrary association. The blue distribution depicts generation 1 while the red distribution depicts the result of 20 generations of selection. All changes from generation 1 to 20 were significant at *p* < 0.05 (independent samples t‐tests) except learning rate for Categorisation.

Figure 5 shows the alteration in the distribution of neurocomputational parameters as a result of selection in each lineage. Selection showed the most dramatic effect on the population frequency of genes affecting numbers of hidden units, and smallest effects on those affecting learning rate.^1^ From the combinations of parameter settings selected, three styles of computation were apparent as the outcome of selection.

First, for categorisation, favoured substrate properties were few hidden units, and high sigmoid temperatures creating sharp category boundaries. Selective pressure did not alter learning rate distributions (independent samples t‐test, *p* > 0.25).

Second, categorisation‐with‐exceptions also selected the high temperature required for sharp category boundaries, but in addition selected increased hidden unit resources. This allowed more internal representational boundaries to accommodate exception patterns. There was a small increase in learning rate (*p* = 0.023).

Third, the remaining domains produced variants of a further computational style with differing degrees of selective pressure, all reflecting the demand to accommodate complex input‐output mappings: a larger number of hidden units, a lower temperature, and a lower learning rate (all *p *< 0.0001). Complex mappings necessitate the integration of multiple constraints captured by combining shallow category gradients provided by many processing units. Such multiple soft constraint satisfaction solutions are best reached by a small learning rate that allows a compromise weights to emerge from repeated exposure to the different mappings of the training set. As complexity increased from quasi‐regular rule to autoassociation to arbitrary association, the selective pressure increased, particularly for hidden units and temperature. For arbitrary association, values approached fixation (the same value across the population), with gene frequencies approaching the highest hidden unit numbers and the lowest temperature values available in the genotype‐phenotype coding scheme.

In sum, divergence in parameter distributions through selection demonstrated the computational underpinnings of domain‐relevance: that acquisition of domains with different computational challenges was optimised by different tuning of otherwise generic neurocomputational properties, such as hidden unit numbers, sigmoid temperature, and learning rate.

Finally, we considered the heritability of individual differences. Formally, heritability is defined as the proportion of variance in the phenotype that is predicted by genetic similarity. It is often estimated using twin designs, which compare the phenotypic similarity of MZ and DZ twins. MZ twins are 100% genetically similar while DZ twins are on average 50% genetically similar. When they are raised in the same family, it is usually assumed they share a common environment (in the simulations, twin networks were trained on the same training set); and therefore, to the extent that the performance of MZ twins is more similar than that of DZ twins, this likely reflects the influence of their greater genetic similarity. Heritability is thus proportional to the difference between MZ and DZ correlations (indeed, following Falconer's equations, under an additive model, it is equal to twice the difference between the MZ and DZ correlations; Falconer 1960).

Table 2 includes the heritability of individual differences for each cognitive domain in generation 1. The table shows the raw difference between MZ and DZ correlations. This value is proportional to the heritability. Exact estimates of heritability are model dependent, requiring an assumption about additivity or dominance of genetic effects, which we wished to avoid here^2^. The statistical reliability of this estimate of heritability was assessed by predicting twin 2 performance from twin 1 performance in a linear regression and testing whether zygosity (MZ or DZ) significantly moderated the correlation when entered as a mean‐centred interaction term. The domain under selection is marked by a box. The shaded areas show domains where performance dropped in the non‐selected domains (see Table 1).

**TABLE 2:** Non‐selected domains can exhibit both reduced performance and higher heritability. Table shows heritability of individual differences in each cognitive domain at Generation 1 and at Generation 20. Outlined boxes indicate the behaviours that were subject to selection in each lineage. Grey shading indicates behaviours showing a decrease in performance across generations (see Table 1).

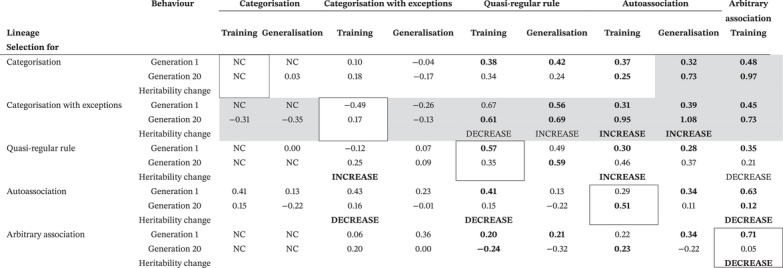

*Note*: Scores show the difference between MZ and DZ correlations. To benchmark the size of these effects, under an additive model, an MZ–DZ correlation difference of 0.5 would correspond to 100% heritability of individual differences. MZ–DZ correlation differences larger than 0.5 imply dominant effects. Bold text indicates MZ–DZ correlation differences reliable at *p* < 0.05, plain text shows trends at *p* < 0.10, greyed text shows non‐reliable differences. Arbitrary association does not embody a generalisable function, therefore performance was assessed on training but not on generalisation. Shaded boxes shows those behaviours where behavioural performance decreases across generations.

INCREASE / DECREASE = change in heritability across generations significant at *p* < 0.05.

Abbreviation: NC, non‐computable (performance at ceiling).

Across the columns, it is evident that heritability depended on the cognitive domain. The computationally easier domains (given the capacity of the ANNs), categorisation and categorisation‐with‐exceptions, did not show the influence of genetic similarity on individual differences due to ceiling effects in performance. The hardest domain, arbitrary association, showed the highest heritability. Heritability of developmental outcomes, then, in part depends on the range of computational processing power in a population compared to the difficulty of the cognitive domains which need to be acquired.

Table 2 includes the change in the heritability of individual differences from generation 1 to generation 20 per lineage and per domain. The reliability of the change in heritability was established testing the 3‐way interaction of generation*zygosity in moderating the twin1‐twin2 correlation. Behaviours under selection are outlined, and behaviours where developmental outcomes declined from generation 1 to 20 are shaded (see Table 1). Behaviours that were selected did not show changes in heritability, except for arbitrary association where it reduced. Figure 5 shows that this is because the parameters for arbitrary association were approaching fixation (most extreme values available) with a consequent reduction in genetic variation in the population. The effects of selection on the heritability of non‐selected behaviours were mixed. Where developmental outcomes declined, in 6 out of 7 cases, heritability increased, although the 2‐way interaction of zygosity and generation was only reliable in two of these cases. The broad pattern suggests that changes in population gene frequency can cause both poorer developmental outcomes for non‐selected behaviours and an increase in heritability of individual differences.

In sum, the second demonstration of the model was that domain‐relevance can not only produce uneven cognitive profiles but also shifts in heritability. Poorer developmental outcomes in specific domains were often linked to raised heritability. The model therefore provides a candidate explanation for the heritable developmental deficits observed in neurodevelopmental conditions, even while genetic influences on the substrate were not themselves specific.

## 4 Discussion

### 4.1 Summary

The goal of the current work was to establish the mechanistic plausibility of the proposal that uneven cognitive profiles, whether in neurodevelopmental conditions or individual differences, may arise from widespread differences in the brain in the neurocomputational properties upon which specific domains particularly rely for their development. We modelled the acquisition of five cognitive domains in different regions of a common substrate. Combining population modelling and evolutionary selection, we showed that altering the frequency of gene variants influencing the neurocomputational properties of the substrate could induce uneven cognitive profiles. Selection for a given behaviour altered the tuning of the computational properties across the substrate, which could impact the development of other skills using different regions of the same substrate. Moreover, these disadvantaged skills could show increased heritability of individual differences in the population.

### 4.2 Limitations

Computational methods are advantageous because they force mechanistic specification of theoretical proposals so that they may be more fully evaluated: are the assumptions of a theory sufficient to explain observed phenomena and under what conditions of implementation? However, these gains are made at the expense of simplification. The novelty of the current work was to employ population‐based modelling and evolutionary selection to link changes in population gene frequencies to uneven cognitive profiles; and twin study designs so that the heritability of uneven behavioural profiles could be considered within a developmental framework, linking gene frequencies to behavioural consequences.

Nevertheless, a range of simplifications was necessary. The cognitive domains were abstract and small scale, rather than naturalistic. The artificial cognitive tasks, while conceptually meaningful, are highly abstracted and may limit the ecological validity and generalisability to real‐world neurodevelopmental conditions. The substrate was modelled by shallow neural networks with at most a few hundred nodes, and only three parameters were varied through selection, restricting biological realism. Populations only numbered 200. Selection was used to drive changes in population gene frequency rather than drift, because drift takes more generations to stochastically alter frequencies. These simplifications were required to train the large number of networks comprising populations over multiple generations in multiple lineages.

At the genetic level, we implemented polygenic influences of gene variants on neurocomputational parameters, but we did not include pleiotropy (the same genes influencing separate biological processes), nor linkage disequilibrium (that nearby genes on chromosomes tend to be inherited together). Implementation of these additional effects would limit the neurocomputational parameter space explored by selection or by drift, since they would reduce the degrees of freedom of parameter variation.

We considered changes in the frequency of common genetic variants exerting polygenic influences on neurocomputational parameters rather than rare variants of large effect or de novo mutations. While this fits with some conditions like ADHD (van der Laan et al. 2025), it is known that other neurodevelopmental conditions like autism have a heterogeneous genetic architecture, and that different sub‐types of the condition, either in phenotypic characteristics or developmental timing, may have different genetic architectures (La Monica et al. 2025; Rylaarsdam and Guemez‐Gamboa 2019; Warrier et al. 2022; Zhang et al. 2025). In other computational modelling work addressing autism specifically, we have exploring how severe variants and combinations of common variants can have similar functional outcomes via common causal pathways influencing neurocomputation (in that case, acting on connectivity; Thomas et al. 2011). We have demonstrated how background risk factors might interact with pathological effects to produce apparent developmental subtypes (the over‐pruning hypothesis; Thomas et al. 2016). This is consistent with recent neuroimaging findings of brain‐wide reductions in neurite density in autism (Gu et al. 2025), despite the uneven cognitive and socioemotional profile that characterises this condition. And it opens up the possibility that part of the genetic heterogeneity in some conditions may be reflecting risk rather than pathological factors.

### 4.3 Theoretical Implications

In her seminal paper which sought to place neurocognitive development at the heart of understanding of neurodevelopmental conditions, Karmiloff‐Smith (1998) rejected domain‐specific explanations of uneven cognitive profiles which appealed to innate modules. She argued that cognitive modularity (functional specialisation) was an outcome of development, and what was then known about genetic influences on brain development did not support specificity at the level of cognition. Emerging theories of functional brain development pointed to the importance of interactivity and competition as drivers of domain specialisation (Johnson 2011; see Mareschal et al. in press, for discussion). It seemed unlikely even then that specific genetic influences on the cortex were involved in sculpting a cognitive ‘theory of mind’ module whose disruption might explain autism, or a ‘phonology’ module whose disruption might explain dyslexia, or a ‘syntax’ module whose disruption might explain developmental language disorder. Nevertheless, the role of genetics is implied by the heritability of these conditions, and a genetic basis of uneven profiles remained to be explicated.

In their review of the development and arealisation of the cortex some two decades later, Cadwell et al. (2019) described 67 genes whose expression has been observed to be enriched in certain cortical areas during development, either in humans or in animal models such as macaque, mouse, rat, or ferret. Many of these genes show gradients of expression along a rostro‐caudal (front to back) axis or medial‐lateral axis. Some show differential expression in lobes, such as frontal, temporal, parietal, or occipital lobes. Some show differential expression in broad functional areas such as somatosensory cortex, visual cortex, or motor cortex. Some show differential expression in a particular layer of a region. There is, then, plentiful evidence of genetic contributions to the regional functional specialisation that emerges across development. However, as Cadwell et al. argue, there is equally evidence that regional gene expression differences are in part influenced by activity dependent processes, and that it is these that lead to the final sharp boundaries between areas of specialised function.

Domain‐relevance provides the missing link that allows more diffuse genetic influences on brain development, say to the temporal lobe, to lead to specific behavioural deficits as the outcome of cognitive development, say in phonological representations that later can support literacy acquisition. However, this idea has required clearer specification to establish what relationships might exist between neurocomputational parameters and the requirements of different domains that support cognitive skills. Previous work applying artificial neural networks to the modelling of neurocognitive conditions such as dyslexia appealed to variation in individual parameters as explanations of deficits, such as neural resources or processing noise (e.g., Harm and Seidenberg 1999). Here we have demonstrated that the computational ‘style’ of the substrate can involve the interaction of multiple parameters: for example, selection for categorisation with exceptions simultaneously altered neural resources, learning rate (plasticity), and activation function (signal to noise).

In sum, the current work supports the domain‐relevance account of the origin of neurodevelopmental conditions, and suggests that evidence of high heritability does not imply genetic influences that are specific to the cognitive domains exhibiting developmental difficulties.

### 4.4 Links to Findings From Genome‐Wide Association Analyses

The finding that combinations of parameters alter the facility of substrate to acquire a cognitive domain gives a ready explanation for why common neurodevelopmental conditions should be highly polygenic, as implied by genome‐wide association studies (GWAS) of dyslexia, developmental language disorder, ADHD, autism, and developmental coordination disorder (Demontis et al. 2023; Doust et al. 2022; Grove et al. 2019; Mountford et al. 2021; Nudel et al. 2023).

Recent findings from GWASs for dyslexia, ADHD, and autism—of larger scale, and therefore greater statistical power to detect DNA variants predicting these conditions—have not shown a direction of travel towards delineating regional or functional specificity. For example, for dyslexia, Doust et al. (2022) identified genes in genotypic loci picked up by the GWAS, and found gene expression was higher in broad brain regions (the cerebellar hemisphere, the cerebellum, and cerebral cortex) but when they investigated 80 structural neuroimaging measures from UK Biobank targeting brain circuitry linked to language, they found no reliable associations. Subsequent amplification of this GWAS (Mountford et al. 2025) similarly only found links to broad regions (e.g., frontal cortex) or cell types (e.g., GABAergic neurons, astrocytes, oligodendrocyte precursor cells). For autism, Gu et al. (2025) found associations between a GWAS‐derived polygenic index and brain‐wide structure indices such as reduced neurite density, reduced cortical surface area, and increased cortical thickness. For ADHD, van der Laan et al. (2025) reported genes linked to GWAS signals were enriched in gene sets differentially expressed in late infancy, enriched across several brain tissue types, and linked to aspects of synaptic function. None of these recent findings points towards the emergence of domain‐specificity between genetic variation and uneven cognitive profiles mediated by specific brain regions or functions. Indeed, genetic correlations with other phenotypes often point to similar genetic basis between neurodevelopmental conditions and general cognitive functions (e.g., Clarke et al. 2016).

### 4.5 Future Hypotheses

Several of the above GWAS have pointed to possible differential genetic effects on brain development at the scale of broad regions like cortical lobes or the cerebellum. The step between these diffuse, large‐scale effects, and domain‐relevant neurocomputational differences within these regions still needs to be articulated. We can speculate on what this account might look like. In the future, rudimentary mapping of neurodevelopmental conditions to broad brain areas or structures may be possible, implicating regional gene expression in heritable components of these conditions (see Thomas and Green 2023, for discussion). For example, if the temporal lobe is more affected, there might be greater impact on language (developmental language disorder, dyslexia), or problems with music; or if the parietal lobe is more affected, there may be greater impact on visuospatial skills (dyscalculia); or if the frontal areas are more affected, there may be greater impact on motor skills (developmental coordination disorder); or if the prefrontal areas are more affected, there may be greater impact on cognitive control skills like maintaining attention and task set (ADHD). Inasmuch as limbic structures are linked to the development of personality, variation in tuning the responses of structures like the amygdala and the insula may be linked to conditions around anxiety (e.g., generalised anxiety disorder), social approach or avoidance behaviour (autism), or empathy (callous and unemotional traits). Variation affecting the interaction of the limbic system with executive functions, such as the orbitofrontal region, may impact emotion regulation (conduct disorder). The basal ganglia are involved in reward‐based action selection. Variation here may be reflected in difficulties with controlling and selecting actions and routines, manifested in conditions like stuttering, obsessive‐compulsive disorder, and Tourette's syndrome. The cerebellum is involved in learning automated motor routines and delivering smooth action. Variation here may produce clumsiness in motor skills and balance (developmental coordination disorder). Rarer conditions affecting the midbrain and brainstem may impact basic sensory systems, such as hearing, or the nerves for sensing the face or controlling eye movements, or autonomic nervous system functions which regulate involuntary physiologic processes including heart rate, blood pressure, respiration, digestion, and sexual arousal.

For these regional effects, we would then need to identify the domain‐relevant computational differences that lead to behavioural variation in functionally specialised regions. Nevertheless, the limitation of a simple regional or system‐by‐system approach is that it predicts clearly differentiated conditions, while disruptions to early developmental processes are likely to impact multiple systems at once. This is particularly seen in genetic syndromes (e.g., Down syndrome; Baburamani et al. 2019; Thomas et al. 2020). But for many behaviourally defined conditions too, the co‐occurrence of multiple difficulties is the rule rather than the exception. High‐level behaviours are delivered by networks of regions across the brain, and disruptions in the connectivity between widespread regions may disrupt development. Alterations in neurotransmitter function may also have widespread impact. Even given more regional effects, developmental interactions between neural systems may compensate for or spread deficits, thereby altering their manifestation in behaviour. Where motivation and reward systems are involved, these may then alter the environments that the child prefers to place themselves in, and therefore the experiences that help shape the self‐organisation of the cortex. It therefore remains a challenge to draw out the pathway from genetic influences at the broader scale through to their final impact on one or more cognitive abilities.

## 5 Conclusion

The main finding of the current computational modelling work was that evolutionary selection for improved performance in one cognitive domain could enhance, impair, or leave unchanged performance in other domains relying on the same substrate depending on their computational demands, thereby producing uneven cognitive profiles—the hallmark of neurodevelopmental conditions. Moreover, domains that showed developmental impairment tended also to show increased heritability, offering a potential explanation for why certain deficits in neurodevelopmental conditions are both uneven and highly heritable. The current work can offer no more than a proof of principle that domain relevance could be the key that links between coarse effects of gene expression on brain development and the specificity observed in uneven cognitive profiles. However, it provides a foundation to build a more plausible framework for understanding links between genotypes and phenotypes in neurodevelopmental conditions, and a stepping stone to unpack the immense biological complexity that lies between the mere fact of heritability of conditions and the developmental pathways through which genetic effects unfold. It also suggests one explanation for why contemporary genome‐wide association studies for neurodevelopmental conditions seem only to pick out more general properties of brain function at the biological level.

## Funding

This work was supported by UK Medical Research Council grant G0300188 awarded to MT. MK's work was supported by a Department of Computer Science and Information Systems studentship at Birkbeck University of London.

## Conflicts of Interest

The authors declare no conflicts of interest.

## Supporting information




**Supporting File 1**: desc70186‐sup‐0001‐SuppMat.docx

**Supporting File 2**: desc70186‐sup‐0002‐tableS6.xlsx

## Data Availability

Simulation code, training sets, and raw data are available on request.

## Endnotes

1 Changes in parameter distributions were also seen for categorisation even though performance was at ceiling accuracy. This is because for truncation selection, the genetic algorithm employed, networks were ranked according to their average mean squared error across the training set. This is a more sensitive measure of performance than accuracy, which was based on thresholding continuously valued activations and computing Hamming distance (see Supplementary Materials).

2 According to Falconer's equations, under an additive model such as ACE, which is valid when (MZ correlation) < 2 x (DZ correlation), heritability is equal to 2(MZ – DZ). Under a dominance model such as ADE, which is valid when MZ < 4 x DZ, the additive component of heritability is (4DZ – MZ) and the dominant component is 2(MZ – 2DZ). In this sense, heritability is model‐dependent based on assumptions about additivity and dominance.

## Technical Implementation Details

### Network Architecture

The substrate for each individual was implemented as a set of five linked three‐layer artificial neural networks sharing the same neurocomputational parameter values but initialised with different randomised weights. The networks were trained independently on the five problem domains using the Rprop algorithm (Riedmiller and Braun 1993). Full implementation details can be found in Supplementary Materials.

### Variation in Neurocomputational Parameters

Three neurocomputational parameters were allowed to vary between individuals, reflecting the influence of genetic variation on neurocomputation: (i) *architecture*: number of hidden units; (ii) *activation dynamics*: slope of logistic activation function within hidden and output units, often called the temperature; and (iii) *adaptation*: the initial learning rate of Rprop. Hidden units affect a network's capacity to learn a training set of a given level of complexity. The steepness of the activation function determines the dynamics acting within each network. Modulation of activation function leads to steeper or shallower slopes in the threshold function of each processing unit. A shallow slope negates the opportunity of the unit to make large changes in its activation level in response to small changes in its input; a steep slope leads to sensitive but binary response characteristics subject to entrenchment effects. Too shallow or too steep values of this parameter may hinder the learning process. Since shallow slopes correspond to a reduction in a unit's sensitivity to changes in its input, it can be considered equivalent to modulation in signal‐to‐noise ratio. The learning rate governs how the network adapts and the speed with which connection weights are altered in response to error signals. Higher rates achieve faster learning on computationally simple mapping problems, but lower learning rates are required to achieve learnability on complex mapping problems which must discover compromise weights that satisfy multiple constraints. However, complexity is defined partly with relation to a network's capacity (technically, the capacity of the network defines the dimensionality of the error surface that the gradient descent algorithm must traverse). The ranges of the parameters were: hidden units: 10–500; temperature: 0.0625–4.0; learning rate: 0.07–0.1.

### Genetic Encoding of Neurocomputational Parameters

The three neurocomputational parameters were encoded into a genome using standard binary representation. Each gene had two variants or alleles, with 10 bits per parameter. Multiple bits per parameter (polygenic coding) were employed firstly to reflect the biological reality of polygenic relationships between protein coding genes and neurocomputational properties such as synaptic function (e.g., Dieterich and Kreutz 2016, found 2000 proteins were involved in the operation of the synapse); and secondly to allow a gradual increase in parameter values, and hence developmental behavioural outcomes, under the pressure of selection, as is observed in animal breeding experiments (e.g., DeFries et al. 1978). The 10‐bit strings were split into two artificial chromosomes for the purposes of sexual reproduction.

A population of ‘parents’ was produced for the first generation of each lineage, by generating random binary vectors for each parent's genotype. Sexual reproduction was used to generate offspring. The 10‐bit string for each parameter was split into two chromosomes. Each parent generated a ‘gamete’ via random selection of one copy of each chromosome to enter the gamete. Two parents, randomly selected, each contributed one gamete with half the genetic information, to create an offspring with the full set of chromosomes. Monozygotic (MZ) twins were created by using the same genotype to instantiate two different networks (although their initial weight randomisation differed). Dizygotic (DZ) twins were created by allowing parents to undergo two breeding events. The genotypes of dizygotic twins therefore had 50% similarity on average. For each generation, 50 pairs of MZ and 50 pairs of DZ twins were produced.

### The Cognitive Domains

Our goal was to identify potential relationships between cognitive domains (modelled here as sets of input‐output mappings) and neurocomputational parameter values. We therefore sought to create training sets with diverse computational challenges and levels of complexity for an artificial neural network.

Assuming each mapping problem is to transform a vector of input values into a vector of output values via one or more intermediate layers of hidden units, and a domain comprises a set of such problems, two related factors influence the computational challenge: (1) whether similar inputs map to similar outputs—the requirement to treat similar inputs in different ways at output is more computationally demanding; (2) the number and mutual consistency of mappings that must be learnt (sometimes referred to as linear separability) across the set; inconsistent mappings are harder to learn, and larger numbers of inconsistent mappings harder still.

Based on these principles, we constructed five problem domains on a scale of easier to harder. The first was *categorisation*, where a single output unit was activated for a binary input pattern, and clusters of similar input patterns activated the same output unit. The second was to add some exceptions to the categorisation problem—a minority of input patterns that fall into different categories to from other patterns to which they are similar. We refer to this as *categorisation with exceptions*. The third domain was a well‐known problem drawn from the field of language development, English past tense formation (see, e.g., Kohli et al. 2020). In this domain, input‐output mappings follow a general rule (form the past tense of a verb by adding ‐ed to its stem) but there are exceptions (e.g., sing‐sang, go‐went). We refer to this domain as a *quasi‐regular rule*. The fourth domain was a general function known as *autoassociation*, where the network must reproduce binary input patterns on the output layer. Although this is a highly consistent mapping, it is demanding in terms of the number of output units which must adopt the correct values. The fifth domain was *arbitrary association*, where the network must learn to map between arbitrary matched random binary patterns. This is the most computationally demanding task. For four of the five tasks, novel items could be generated that fell into existing categories, or to which the rule (past tense, autoassociation) could be applied. For these problems, novel datasets were created to test generalisation. Each problem was instantiated over 57 input units and 62 output units, and training sets comprised 500 input‐output mappings. Full details on the construction of the training and test sets can be found in Supplementary Materials.

### Variation in the Environment

A filter was used to alter the quality of the training sets and so produce variations in cognitive stimulation (Rakesh et al. 2024). An individual's environmental quality was modelled by a number selected at random from the range 0.6–1.0. This gave a probability that any given pattern in the full training set for each domain would be included in that individual's training set. The range 0.6–1.0 ensured that all individuals were exposed to more than half of the training dataset. Performance was always tested on the full training set. The approach of filtering training sets has been used to model environmental influences on language development, such as those associated with differences in socioeconomic status (see, e.g., Thomas et al. 2013; Thomas and Coecke 2023). Similarly, we took environmental quality to apply to the family rather than the individual, so that twin pairs were trained on the same filtered training set.

### Evolutionary Selection

For each generation, at the end of training, individuals were ranked on their performance on the target cognitive domain under selection. For each twin pair, one was designated to be in the breeding population. Individuals were chosen for breeding using truncation selection, with only the top 50% of performing individuals allowed to breed. Selected individuals were randomly paired to generate the next generation using sexual reproduction. Selection operated for 20 generations. This value was determined based on a preliminary experimental study with one of the datasets, where 5, 10, 15 and 20 generations were evaluated. The objective was to ensure there was no premature convergence, a situation where the population loses genetic diversity too early, thereby reducing the algorithm's ability to explore the search space and generate better offspring and potentially leading to suboptimal solutions. Satisfactory convergence was observed after 15 generations. However, 20 generations were ultimately selected to promote sustained exploration and ensure the algorithm reached a steady state with potentially higher‐quality solutions across all datasets.

### Simulation Design

Five lineages were created each starting with 200 individuals, comprising 50 MZ twin pairs and 50 DZ twin pairs. The first generation was produced from parents with randomly initialised artificial genomes. In each lineage, one of the five problem domains was the target domain for selection, although the five linked networks were always trained on the five domains. Networks were trained for 500 epochs. Selection operated for 20 generations. The simulations, therefore, comprised 20,000 sets of five linked 3‐layer networks, 100,000 networks overall.
