# Supplementary material for: The Genetic Origin of Uneven Cognitive Profiles in Heritable Neurodevelopmental Conditions and Individual Differences: Computational Investigations
Source: Dev Sci. 2026 Apr 16;29:e70186. doi: 10.1111/desc.70186 (PMC13084292; doi:10.1111/desc.70186)
Supplement: Supplementary file 1 — Supporting File 1: desc70186‐sup‐0001‐SuppMat.docx [file DESC-29-e70186-s001.docx]

**Supplementary materials for:**

**The genetic origin of uneven cognitive profiles in heritable neurodevelopmental conditions and individual differences: Computational investigations**

Maitrei Kohli^1^ (maitrei.kohli@ucl.ac.uk)

George Magoulas^2^ (g.magoulas@bbk.ac.uk)

Michael S. C. Thomas^3^* (m.thomas@bbk.ac.uk)

* corresponding author

^1^ Department of Computer Science, University College London

^2^ School of Computing and Mathematical Science, Birkbeck University of London

^3^ Developmental Neurocognition Lab, Centre for Brain and Cognitive Development, School of Psychological Sciences, Birkbeck University of London

Corresponding author:

Professor Michael S. C. Thomas

Centre for Brain and Cognitive Development

School of Psychological Sciences

Birkbeck University London

Malet Street, Bloomsbury

London WC1E 7HX, UK

Email: [m.thomas@bbk.ac.uk](mailto:m.thomas@bbk.ac.uk)

Orcid: <https://orcid.org/0000-0002-8231-6011>

**Supplementary Materials**

**Methods**

*Network training*

Experiments were conducted on Condor, which is a platform that supports running high throughput computing on large collections of distributive owned computing resources (Thain et al., 2005). It follows a master-slave type configuration, which has proved suitable for training neural network architectures (Plagianakos et al., 2006).

The simulations involved training 100,000 shallow 3-layer artificial neural networks (ANNs) on five different training sets. Five linked networks comprised each individual’s modelled substrate. For efficiency, networks were trained using the batch version of the Rprop algorithm (Riedmiller & Braun, 1993) with an error goal (mean squared error) of 10^-5^, a maximum number of epochs (either 100 or 500 depending on complexity of the training set) and an early stopping condition were training stopped where if the accuracy had not improved for 20 epochs (50 epochs for more complex training sets) training ceased and the algorithm returned the best epoch performance.

To ensure fast convergence, weight initialisation used random values across the interval $[-\frac{a}{\surd din},+\frac{a}{\surd din}$] wherein $a$ is chosen in a way that weight variance corresponds to the points of maximum curvature of activation function. This value is $2.38$ for standard sigmoid function (Thimm & Fiesler, 1995); and $d_{in}$ is fan-in of neuron or the total number of inputs of a neuron in the network.

Performance of each network was assessed on the full training set for each domain and where appropriate, also on a novel dataset created to test the generalisation ability of the networks. Accuracy was measured by converting continuous output values to binary values by applying a threshold of 0.5 and then computing Hamming distance. Where all bits matched the target, mean squared error was also computed between continuously valued outputs and binary targets, so that a rank order of performance could be computed for networks which scored identically on accuracy.

*Variation in network parameters*

Three neurocomputational parameters were allowed to vary, reflecting the influence of genetic variation on neurocomputation. The three parameters were: (i) architecture: number of hidden units; (ii) activation dynamics: slope of logistic activation function within hidden and output units; and (iii) adaptation: the initial learning rate of Rprop.

Hidden units affect how a network is built and thus relate to network’s capacity to learn a training set of a given level of complexity. The steepness of the activation function corresponds to the activation dynamics acting within each network. The transfer function is an important part of ANN architecture and has significant impact on its performance (Yao, 1999). Modulation of activation function leads to steeper or shallower slopes in the threshold function. A shallow slope negates the opportunity of a processing unit to make large output changes in response to small changes in input; a steep slope ultimately leads to very sensitive but binary response characteristics subject to entrenchment effects. Therefore, too shallow or too steep values of this parameter will hinder the learning process (Plagianakos et al., 2006; Thomas et al., 2016). Since shallow slopes correspond to a reduction in a unit’s ability to reflect small differences in its input as changes in its output, it can be considered equivalent to a change in signal to noise ratio. Heuristic learning parameters, such as the learning rate, govern how the network adapts and hence provide a network with the ability to learn.

The range of variation of the three parameters was calibrated to avoid the presence of parameter values in the population that produced networks with no learning ability at all. The five training sets, in order of increasing computational complexity, were categorisation, categorisation with exceptions, quasi-regular rule, autoassociation, and arbitrary association (see below). The middle of these, quasi-regular rule, was used to calibrate the range of variation across which the three parameters could vary in populations. Calibration was achieved by beginning with random values for all parameters and training 100 neural networks for 1000 epochs while varying the values, in steps of 5 for hidden units and 0.01 otherwise, for each of these parameters individually. The calibration process was carried out for all parameters, until values were identified beyond which the learning failed, as well as the values which resulted in increasingly successful learning. The resulting parameter ranges are shown in Table S1.

**Table S1**: parameter ranges allowed in the populations of artificial neural networks

| Neuro-computational Parameters | Type of parameter | Range of Variation |
| --- | --- | --- |
| No. of hidden units | Structural | 10 - 500 |
| Initial learning rate | Learning | 0.07 – 0.1 |
| Slope of logistic activation function | Node transfer | 0.0625 – 4.0 |

*Encoding network parameters into an artificial genome*

The three neurocomputational parameters were encoded into a genome using standard binary representation. This allowed populations to be created with variable parameter ranges using a scheme that could both encode genetic relatedness (specifying identical and non-identical twins) to allow calculations of heritability, and also permit selection of adaptive gene variants through sexual reproduction. The genotypes were constructed as concatenated binary strings. Each gene had two variants or alleles, with 10 bits per parameter. Multiple bits per parameter (so-called *polygenic coding*) was used firstly to reflect the biological reality of polygenic relationships between protein coding genes and neurocomputational properties such as synaptic function (e.g., Dieterich & Kreutz, 2016), and secondly to allow a gradual increase in parameter values, and hence developmental behavioural outcomes, under the pressure of selection, as is observed in animal breeding experiments (e.g., DeFries et al., 1978). The 10-bit strings were split into two artificial chromosomes for the purposes of sexual reproduction. Figure S1 gives an example of genome.

**Figure S1**: An example artificial genome encoding the three neurocomputational parameters

| $\boldsymbol{Genome}$ | $\boldsymbol{1 1 1 0 0 1 0 1 0 1}$ | $\boldsymbol{0 0 1 1 0 1 1 0 0 0}$ | $\boldsymbol{1 0 1 1 0 0 1 1 0 0}$ |
| --- | --- | --- | --- |
|  | $HU$ | $LR$ | $Slope$ |

The range of variation of the parameter values established by the calibration phase then served as the upper and the lower bound used for converting the genotype (binary encoded values) into its corresponding phenotype (real valued neurocomputational parameters). There are number of ways in which binary-to-real conversions can be carried out. In this work, Matlab genetic Algorithm Toolbox (http://codem.group.shef.ac.uk/index.php/ga-toolbox) was using, including the library function called $bs2rv$ which has a decoding matrix. This matrix had the following parameters to accomplish this binary to real conversion – length of each binary string ($len$); lower and upper bounds for each encoded gene (neuro-computational parameter) ($lb$ and$ub$); type of encoding –binary or grey ($code$); type of scaling to be used for each string – arithmetic or logarithmic ($scale$); and finally whether or not to include the lower and/or upper bound in the representation range ($lbin$ and$ubin$). As an example, consider a population consisting of three ANNs with their neuro-computational properties of number of hidden units ($HU$), learning rate ($LR$) and the slope of logistic activation ($Slope$) encoded in genotype ($G$), with each gene having four bits:

$$\boldsymbol{HU}$$

$$\boldsymbol{LR}$$

$$\boldsymbol{Slope}$$

$$G=\left[ \begin{aligned} 0 0 1 1 0 1 1 1 1 1 0 0 \\ 1 0 0 0 1 0 0 1 0 0 0 1 \\ 0 0 1 0 1 0 0 0 0 0 1 1 \end{aligned} \right]$$

Let the decoding matrix (known as$FieldD$) based on the range given in Table S1 be specified according to the values shown in Table S2:

**Table S2**: specification of the decoding matrix

| $\boldsymbol{fieldD=}$ | $\boldsymbol{4 4 4}$ | $\boldsymbol{10 0.07 0.0625}$ | $\boldsymbol{500 0.1 4.0}$ | $\boldsymbol{0 0 0}$ | $\boldsymbol{1 1 1}$ | $\boldsymbol{1 1 1}$ | $\boldsymbol{1 1 1}$ |
| --- | --- | --- | --- | --- | --- | --- | --- |
|  | $Len$ | $Lb$ | $Ub$ | $Code$ | $Scale$ | $Lbin$ | $Ubin$ |

The decoding matrix specifies the length of each gene, the lower and upper bounds for the range, specifies binary coding (0 represents binary and 1 grey), arithmetic scaling (1 represents arithmetic and 0 logarithmic) and finally that the lower and upper bounds are included in representation range as depicted by ones (otherwise use zero to exclude them from range). Therefore, the resulting phenotype, $Phen$ will be a function of $G$ and$fieldD$, i.e.

$$Phen = f(G, fieldD)$$

For this example, the resulting three ANNs will have the parameter values shown in Table S3:

**Table S3**: example parameter values for three ANNs

| $\boldsymbol{Phen =}$ | $\boldsymbol{HU}$ | $\boldsymbol{LR}$ | $\boldsymbol{Slope}$ |
| --- | --- | --- | --- |
|  | $22$ | $0.082$ | $1.74$ |
|  | $81$ | $0.086$ | $0.08$ |
|  | $17$ | $0.084$ | $0.14$ |

Each individual’s 30-bit genome was therefore converted into three parameter values, which were common across the five linked ANNs that comprised each individual’s modelled substrate. Note that network weights were not encoded in genome to be evolved. Instead, weights were always initially randomised in each network, and continuously modified during the lifetime via training. In this way, genetically inherited information influencing the neurocomputational parameters interacted with information coming from the environment external to the network to establish its weights through a developmental process (Nolfi & Floreano, 1999).

*Sexual reproduction and creating populations of twins*

A population of ‘parents’ was produced for the first generation of each lineage, by generating random binary vectors for each parent’s genotype. Sexual reproduction was used to generate offspring. The 10-bit string for each parameter was split into two chromosomes. Each individual generated a ‘gamete’ via random selection of one copy of each chromosome to enter the gamete. Crossover events were permitted, allowing recombination of the two chromosomes before the single copy entered the gamete (see Kohli, 2020). Six crossover events were used in these simulations. Two parents, randomly selected, each contributed one gamete with half the genetic information, to create an offspring with the full set of chromosomes. Monozygotic (MZ) twins were created by using the same genotype to instantiate two different networks (although their initial weight randomisation differed). Dizygotic (DZ) twins were created by allowing parents to undergo two breeding events. The genotypes of dizygotic twins would therefore have 50% similarity on average.

For each generation, 50 pairs of MZ and 50 pairs of DZ twins were produced. For evolutionary selection, twins were split in breeding and non-breeding sets, where the former was the population containing the first twin out of each of the twin pairs (100 networks) and the latter was the population containing the remaining second twin of a twin pair (100 networks). Therefore, only one twin from each pair contributed genes to the following generation.

*Evolutionary selection*

For each generation, at the end of training, individuals were ranked on their performance on the target domain under selection. Selected individuals were randomly paired to generate the next generation using sexual reproduction. There are several selection methods used in genetic algorithms. We employed truncation selection, which is an efficient form of directional selection. In this method, only the fittest X% of the population are selected as parents for breeding, where X can usually take any value from 50% - 100%. Individuals below this fitness threshold do not get chosen for breeding at all (in other methods, such as roulette-wheel based selection, breeding has a probabilistic relationship to fitness). The main advantage of truncation selection is rapid convergence, the potential disadvantage is possible local minima. For our simulations, X=50%, so that only the top half of the population was allowed to breed.

*Training sets*

Because our focus was to identify potential relationships between cognitive domains (modelled here as sets of input-output mappings) and neurocomputational parameter values, we sought to create training sets with diverse computational challenges and levels of complexity for an artificial neural network. Assuming each problem is to transform a vector of input values into a vector of output values via one or more intermediate layers of hidden units, and a domain comprises a set of such mappings, two related factors influence the computational challenge: (1) whether similar inputs map to similar outputs – the requirement to treat very similar inputs in different ways is more computationally demanding; and (2) the number and mutual consistency of mappings that must be learn (the latter sometimes referred to as linear separability); inconsistent mappings are harder to learn, and larger numbers of inconsistent mappings harder still.

Based on these factors, we constructed five problem domains on a scale of easier to harder. The first was categorisation, where a single output unit was activated for a binary input pattern, and clusters of similar input patterns activated the same output unit. The second was to add some exceptions to the categorisation problem – a minority of input patterns that fall into different categories to the other patterns to which they are similar. The third domain was a well-known problem drawn from the field of language development, English past tense formation. Input-output mappings follow a general rule (form the past tense of a verb by adding -ed to its stem) but there are sets of exceptions (e.g., sing-sang, go-went). Here we will refer to this as a quasi-regular rule. The fourth domain was a general function known as autoassociation, where the network must reproduce binary patterns from the input on the output layer. This is demanding in terms of the number of output units which must adopt the correct values, but is a highly consistent mapping. The fifth domain was arbitrary association, where the network must learn to map between arbitrarily linked random binary patterns. This is the most demanding computationally. For four of the tasks, novel items could be generated that fell into existing categorises, or to which the rule (past tense, autoassociation) could be applied. For these problems, novel sets were created to test generalisation. Each problem was instantiated over 57 input units and 62 output units. Details of the training sets are described below and summarised in Table S4.

Piloting suggested autoassociation and arbitrary association were more demanding problems, so they were trained for 500 epochs with a stopping condition of 50 epochs. Categorisation, categorisation with exceptions, and quasi-regular rule were trained for 100 epochs with a stopping condition of 20 epochs.

*Categorisation:* The training set for the categorisation task considered the assignment of input patterns to ten categories, based on their similarity to a prototype pattern for each category. The training set was created by first defining the ten prototype patterns to define each category. These were ten random vectors of 57 binary digits. Next, clusters of input patterns similar to the prototypes were generated. Each input pattern was created by altering each bit of corresponding prototype pattern with a probability of 0.05. Any duplicates produced were discarded. To encode categories in the output layer of the network architecture, the 60 leftmost output units were used. Ten groups of six units were considered, each group corresponding to one category. When an input pattern belonged to a certain category the units of the corresponding group of output units had the value of 1. Training set contained 500 patterns.

A generalisation dataset was also used to evaluate the ability of the networks to categorise novel items based on their similarity to the prototype patterns. The input patterns of the generalisation set were constructed with the same procedure followed for training set generation. Bits of the prototype vectors were altered probabilistically, with any resulting duplicates being removed from generalisation dataset. Generalisation dataset had 500 patterns.

*Categorisation with Exceptions*: The training set for this task used the same input patterns as the categorisation training set. However, this version corresponded to a slightly more complex categorisation problem, which additionally considered a sub-cluster of exceptions. This sub-cluster consisted of all input patterns of category 9 whose Euclidean distance from the prototype element was less than 2. The sub-cluster of these input patterns should be assigned to category 7, instead of category 9. The generalisation set was implemented for this task with the same methods used for categorisation task, but which also included the sub-cluster of exceptions. There were 500 patterns in training and generalisation dataset respectively and 10 patterns belonged to the sub-cluster.

*Quasi-regular rule*: This problem was drawn from the literature which has applied artificial neural network models to the simulation of language acquisition and in particular the domain of English Past Tense. The dataset was based on the “phone” vocabulary from the Plunkett and Marchman (1991) past-tense model. The past tense domain is modelled by an artificial language created to capture many of the important aspects of the English language, while retaining greater experimental control over the similarity structure of the domain (Plunkett and Marchman, 1991). The dataset comprised of artificial verbs which in effect were artificial monosyllabic phoneme strings that followed one of three templates – CCV, VCC, and CVC (where C is a consonant and V is a vowel). There were 508 verbs in the dataset. Each verb had three phonemes – initial, middle, and final. The phonemes were represented over 19 binary features using an encoding based on linguistic articulatory features (Thomas and Karmiloff-Smith, 2003). A network thus had 3×19 = 57 input units and 3×19 + 5 = 62 units at the output. The extra five units in the output layer were used for representing the affix for regular verbs in binary format.

In the training dataset, there are 410 regular and 98 irregular verbs. These were further be divided into four types: regular verbs that form their past tense by adding /ed/ - e.g. talk – talked; regular verbs which form past tense by adding /d/ - e.g. tame – tamed, regular verbs which suffix /t/ - e.g. send – sent and finally the irregular verbs, e.g. hide – hid or go – went. In the dataset, out of 410 regulars, there were 271 /ed/ verbs, 90 /d/ verbs, 49 /t/ verbs.

A second dataset was used to assess the generalisation performance of the model. The main intent was to measure the degree to which an ANN can reproduce in the output layer properly inflected novel items presented in the input. The generalisation set comprises 410 novel verbs, each of which shared at least two phonemes with one of the regular verbs in the training set, for example wug => wugged (Thomas et al., 2016).

*Auto-association*: The input patterns of the training set for this task were random vectors of 57 binary digits. The target patterns were same as the input patterns. As the architecture of the network had 62 units in the output layer, the targets were presented in the 57 leftmost units of the output layer. The remaining five units had zero values for all mappings. There were 500 patterns in the training dataset.

A generalisation dataset was also constructed by probabilistically altering the patterns of the training set. Each bit of input pattern was flipped (from 1 to 0 or vice versa) with a probability of 0.2. If the resulting pattern was not novel the procedure was repeated. There were 500 patterns in the generalisation dataset.

*Arbitrary association*: The training set for arbitrary mappings task had the same input patterns as the training set for auto-association task. The output patterns were random vectors of 62 binary digits. Because the mappings were random, there was no underlying systematic function. Therefore, there was no generalisation dataset to test the extension of the function. There were 500 patterns in the dataset.

**Table S4**. Summary of the five problem domains

| Tasks | Input Bits | Output Bits | Description of data set |
| --- | --- | --- | --- |
| Categorisation | 57 | 62 | 1. Training set consists of 500 patterns belonging to 10 categories 2. Each pattern is assigned a category based on its similarity to the prototype pattern of each category. 3. Each pattern is created by altering each bit of corresponding prototype pattern with a probability of 0.05. 4. Test set consisting of 500 novel patterns using the same procedure used for training set. |
| Categorisation with exceptions | 57 | 62 | 1. Training set consists of 500 patterns where same input patterns as in previous categorisation data set are used. 2. Slight modification in the mappings. Includes a sub cluster of exceptions. 3. This sub cluster consists of all input patterns of category 9 whose Euclidean distance from prototype element of the category is less than 2. 4. These patterns are assigned category 7, instead of 9.   Test set consisting of 500 novel patterns using the same procedure used for training set. |
| Quasi-regular rule | 57 | 62 | 1. The training set consists of 500 English past tense verbs type frequency of verbs: 410 regular verbs (stem => stem+ed), 20 exception no-change verbs, 68 exception vowel change verbs, and 10 exception arbitrary verbs 2. Separate test set consisting of 410 novel verbs rhyming with existing regular verbs |
| Autoassociation | 57 | 62 | 1. Training set consists of 500 patterns and Target patterns same as input patterns 2. ANNs produce 62-bit output vectors (62 output nodes) but the last 5 bits get zero values for all mappings. 3. Separate test set consists of 500 novel patterns. |
| Arbitrary Association | 57 | 62 | 1. Training set consists of 500 random binary vectors; targets are unrelated random binary vectors 2. No generalisation set since random inputs have random outputs |

*Variation in training sets*

Assessments of heritability – the contribution of genetic variation to predicting phenotypic variation – require there to be environmental sources of variation which also influence development of the phenotype. This was implemented by altering the quality of the training sets by applying a filter. An individual’s environmental quality was modelled by a number selected at random from the range 0.6-1.0. This gave a probability that any given pattern in the full training from each domain would be included in that individual’s training set. The range 0.6-1.0 defined the range of variation of environmental quality, and ensured that all individuals were exposed to more than half of the training dataset, i.e., had a decent view of the problem domain. Performance was nevertheless always tested on the full training set. The approach of filter training sets has been used to model environmental influences on language development, such as those associated with differences in socioeconomic status (see, e.g., Thomas, Forrester & Ronald, 2013; Thomas & Coecke, 2023). Because we modelled twin pairs raised in the same family, in line with the twin method usually used to assess heritability, each ‘twin’ network was trained on the same filtered training set, so that the environmental quality was taken to apply to the family rather than the individual.

A summary of the simulations is shown in Table S5.

**Table S5**. Summary of simulations

| No. of lineages | 5 |
| --- | --- |
| No of Generations per lineage | 20 |
| Size of population | Breeding = 100  Non-breeding = 100  Total of 5 lineages across 20 generations = 100,000 ANNs or 20,000 linked sets of 5 networks depicting each substrate |
| Size of Datasets | Training = 500 {508 for quasi-regular rule}  Generalisation = 500 |
| Training Mode | Batch |
| Max. training epochs | 100 (Categorisation, Categorisation with exceptions, Quasi-regular rule)  500 (Autoassociation, Arbitrary association) |
| Early Stopping Criterion, maxstep (i.e. stop training if training accuracy does not improve till step == maxstep) | 20 (Categorisation, Categorisation with exceptions, Quasi-regular rule)  50 (Autoassociation, Arbitrary association |
| Initial weight update (Rprop learning rate), delta0 | Values from genome |
| Hidden units. Steepness of logistic | Values from genome |
| Selection Operator | Truncation – 50 top performing breeding networks chosen at the end of training |
| Source Task for Selection | Categorisation, Categorisation with exceptions, Quasi-regular rule, Autoassociation, Arbitrary association |
| Crossover | 6 crossovers/chromosome; single-point, multi-point & shuffle operators used |
| Environmental Factor | Probability value between 60% and 100% |
| Range of encoded neurocomputational parameters | No. of hidden units (10 – 500); initial learning rate (0.7 – 1.0); slope of logistic activation (0.0625 – 4.0) |

**Results**

Table S6 shows the correlations between MZ and DZ twin pairs at the end of training for each lineage and each problem domain. Heritability is proportional to the difference between MZ and DZ correlations (following Falconer’s equations, under an additive model it is equal two twice the difference). In Table S6, we show the raw difference between MZ and DZ correlations, which avoids the need to make assumptions about additivity or dominance of genetic effects, and this value can be taken as proportional to the heritability. The statistical reliability of these heritability measures was assessed by predicting twin 2 performance from twin 1 performance in a linear regression, and then testing whether zygosity (MZ or DZ) significantly moderated the correlation when entered as a mean-centred interaction term. Heritabilities are shown for generation 1 and for generation 20. The reliability of the change in heritability was established by including generation as a variable and testing the 3-way interaction of generation*zygosity in moderating the twin1-twin2 correlation (again, interaction terms were entered with mean-centred variables).

**Table S6**. Heritabilities for performance on each domain in each lineage, comparing the first generation and the 20^th^ generation. The highlighted box shows heritabilities in each lineage for the domain under selection. Values are omitted for cases where performance was at ceiling and so there was no variability to compute a correlation.

**References**

DeFries, J. C., Gervais, M. C., & Thomas, E. A. (1978). Response to 30 generations of selection for open-field activity in laboratory mice. *Behav Genet*. 1978 Jan;8(1):3-13. doi: 10.1007/BF01067700.

Dieterich, D. C., & Kreutz, M. R. (2016). Proteomics of the Synapse--A Quantitative Approach to Neuronal Plasticity. *Mol Cell Proteomics*. 2016 Feb;15(2):368-81. doi: 10.1074/mcp.R115.051482.

Kohli, M. (2020). *Evolving Neural Networks Using Behavioural Genetic Principles*. Unpublished PhD Thesis, Birkbeck University of London. https://www.dcs.bbk.ac.uk/site/assets/files/1025/mkohli.pdf

Nolfi, S., & Floreano, D. (1999). Learning and evolution. *Autonomous robots*, 7(1), 89-113.

Plagianakos, V. P., Magoulas, G. D., & Vrahatis, M. N. (2006). Evolutionary training of hardware realizable multilayer perceptrons. *Neural Computing & Applications*, 15(1), 33-40.

Plunkett, K., & Marchman, V. (1991). U-shaped learning and frequency effects in a multi-layered perception: Implications for child language acquisition. *Cognition*, 38(1), 43-102.

Riedmiller, M., & Braun, H. (1993). A direct adaptive method for faster backpropagation learning: The RPROP algorithm. In *Neural Networks, 1993., IEEE International Conference On* (pp. 586-591). IEEE.

Thain, D., Tannenbaum, T., & Livny, M. (2005). Distributed computing in practice: the Condor experience. *Concurrency and computation: practice and experience*, 17(2‐4), 323-356.

Thimm, G., & Fiesler, E. (1995): Neural network initialization. From Natural to Artificial Neural Computation. *Lecture Notes in Computer Science Volume 930* (pp.535-542).

Thomas, M. S. C. & Coecke, S. (2023). Associations between Socioeconomic Status, Cognition, and Brain Structure: Evaluating Potential Causal Pathways Through Mechanistic Models of Development. *Cogn Sci*. 2023 Jan;47(1):e13217. doi: 10.1111/cogs.13217.

Thomas, M. S. C., Forrester, N. A., & Ronald, A. (2013). Modeling Socioeconomic Status Effects on Language Development. *Developmental Psychology*, 49(12), 2325-43. doi: 10.1037/a0032301

Thomas, M. S. C., Forrester, N. A., & Ronald, A. (2016). Multiscale modeling of gene–behavior associations in an artificial neural network model of cognitive development. *Cognitive Science*, 40(1), 51–99. <https://doi.org/10.1111/cogs.12230>

Thomas, M. S. C., & Karmiloff-Smith, A. (2003). Modeling language acquisition in atypical phenotypes. *Psychological Review*, 110(4), 647–682. https://doi.org/10.1037/0033-295X.110.4.647

Yao, X. (1999). Evolving artificial neural networks. *Proceedings of the IEEE*, 87(9), 1423-1447.
